# Effect of Al_2_O_3_ (x = 0, 1, 2, and 3 vol.%) in CrFeCuMnNi-x High-Entropy Alloy Matrix Composites on their Microstructure and Mechanical and Wear Performance

**DOI:** 10.3390/ma16103672

**Published:** 2023-05-11

**Authors:** S. Sivasankaran, Hany R. Ammar, El-Sayed M. Sherif, Abdulaziz S. Alaboodi, Abdel-baset H. Mekky

**Affiliations:** 1 Department of Mechanical Engineering, College of Engineering, Qassim University, Buraydah 51452, Saudi Arabia; 2 Center of Excellence for Research in Engineering Materials (CEREM), Deanship of Scientific Research, King Saud University, Riyadh 11421, Saudi Arabia; 3 Department of Physics, College of Science and Arts El-Meznab, Qassim University, Buraydah 51931, Saudi Arabia

**Keywords:** high-entropy alloys, Al_2_O_3_, X-ray diffraction, microstructures, mechanical, wear

## Abstract

This work aims to study the influence of Al_2_O_3_ in CrFeCuMnNi high-entropy alloy matrix composites (HEMCs) on their microstructure, phase changes, and mechanical and wear performances. CrFeCuMnNi-Al_2_O_3_ HEMCs were synthesized via mechanical alloying (MA) followed by hot compaction (550 °C at 550 MPa), medium frequency sintering (1200 °C), and hot forging (1000 °C at 50 MPa). The XRD results demonstrate the formation of both FCC and BCC phases in the synthesized powders, which were transformed into major stable FCC and minor ordered B2-BCC phases, as confirmed by HRSEM. The microstructural variation of HRSEM-EBSD, in terms of the coloured grain map (inverse pole figures), grain size distribution, and misorientation angle, was analysed and reported. The grain size of the matrix decreased with the increase in Al_2_O_3_ particles owing to the higher structural refinement by MA and zener pinning of the incorporated Al_2_O_3_ particles. The hot-forged CrFeCuMnNi-3 vol.% Al_2_O_3_ sample exhibited an ultimate compressive strength of 1.058 GPa, which was 21% higher than that of the unreinforced HEA matrix. Both the mechanical and wear performance of the bulk samples increased with an increase in Al_2_O_3_ content due to solid solution formation, high configurational mixing entropy, structural refinement, and the effective dispersion of the incorporated Al_2_O_3_ particles. The wear rate and coefficient of friction values decreased with the increase in Al_2_O_3_ content, indicating an improvement in wear resistance owing to the lower domination of abrasive and adhesive mechanisms, as evidenced by the SEM worn surface morphology.

## 1. Introduction

Recently, high-entropy alloys (HEAs) have attracted the attention of the research community owing to their excellent mechanical, wear, thermal, fatigue, and surface properties. HEAs are a multicomponent solid solution category that usually possesses complicated microstructures due to the lack of restrictions on the alloy design. HEAs retain mechanical properties (strength and hardness) at elevated temperatures and are less susceptible to corrosion [1,2,3]. According to Yeh et al. [4], HEAs can be designed and developed by mixing five or more metallic elements in equiatomic or near equiatomic ratios, which introduces a high-configurational entropy effect in materials. High configurational entropy (promoting stable solid solution), sluggish diffusion (controlling microstructure and enhancing creep resistance), severe lattice distortion (promoting solid solution hardening and thermal resistance), and cocktail effects (controlling phase formation and promoting hardness) are the four effects that alter the microstructure and mechanical properties of HEAs [5,6,7,8].

The incorporation of ceramic particles (oxides, carbides, and nitrides) into HEAs can further enhance their mechanical properties and be applied to industrial and structural parts [9,10]. Generally, HEAs exhibit single-phase FCC or BCC or a combination of FCC/BCC/HCP phases (multi-phase alloys) [11,12,13,14]. The addition of ceramic particles to HEAs causes the production of more interfaces, dislocation loops, and voids in the grain boundaries, which are expected to promote a hardening effect in the structure [15]. Furthermore, maintaining a stable phase is a major challenge in HEAs because of grain growth at elevated temperatures, which can be eliminated by incorporating ceramic particles [16]. Hadraba et al. [17] developed CoCrFeNiMn-0.3 wt.% Y_2_O_3_ high-entropy alloy matrix composites (HEAMCs) by an in situ reaction technique. The Al_0.3_CoCrFeMnNi-0.3 Y_2_O_3_ HEAMC was recently developed and investigated by Gwalani et al. [18]. The HEAMC produced 1800 MPa of mechanical strength, which was 80% higher than the Al_0.3_CoCrFeMnNi HEA matrix. Prasad et al. [19] synthesised FeNiCoCrAlMn reinforced with 2 wt.% of Al_2_O_3_ HEAMC by the mechanical alloying (MA) route and studied the thermal behaviour. Their results indicated that the developed sample exhibited a higher resistance to temperature and oxygen. CrMnFeCoNi-0.25 wt.% Y_2_O_3_ HEAMC was synthesized by Liu et al. [20], who studied the tensile and wear properties. Jia et al. [21] developed an FeCrCoNi-5 wt.% Y_2_O_3_ HEAMC through the MA route, and studied the microstructure and mechanical behaviour. Yang et al. [22] developed Al_0.4_FeCrCo_1.5_NiTi_0.3_-8 vol.% Al_2_O_3_ HEAMC and achieved a single-phase FCC structure.

Based on the literature, it can be observed that improved strength and ductility can be achieved by the addition of ceramic particles into the HEA matrix, which produces high-entropy alloy matrix composites (HEAMCs) [23]. HEAMCs can be manufactured via vacuum arc melting (VAM [24]), mechanical alloying and spark plasma sintering (MA-SPS [25,26]), induction melting (IM [27]), MA and hot-isostatic pressing (MA-HIP [9,28]), vacuum hot-pressing (VHP [29]), fusion deposition (FD [30]), and laser surface injection (LSI [31]). Al_2_O_3_, Y_2_O_3_, WC, TiC, TiN, and SiC [32] are commonly used ceramic particles in HEA that extensively alter the matrix properties. Based on the literature, it is evident that the addition of oxide particles successfully alters the mechanical and structural properties of HEAs [23,28,31,32]. Furthermore, there has been no study on the development and investigation of CrFeCuMnNi HEA matrices reinforced with different vol.% of ultra-fine Al_2_O_3_ particles (105 nm).

Therefore, the present study is conducted because of the limited number of studies on the synthesis of HEAMC powders of CrFeCuMnNi-x vol.% Al_2_O_3_ (x = 0, 1, 2, and 3) by MA. The synthesized nanostructured powders were consolidated using hot compaction, followed by medium-frequency sintering and hot pressing. The synthesized powders and bulk samples were characterized by XRD, HRSEM with EDAX, and EBSD analyses. The relative density, mechanical properties (in terms of compressive stress–strain curves, ultimate compressive strength, and strain at the ultimate point), and wear behaviour by the sliding wear test of the prepared bulk samples with the function of Al_2_O_3_ reinforcements are investigated and reported. Finally, this study recommends the developed HEAMCs for use in aerospace and automotive parts.

## 2. Materials and Methods

### 2.1. Synthesis of CrFeCuMnNi-Al_2_O_3_ HEAMCs

To synthesise CrFeCuMnNi- x vol.% Al_2_O_3_ (x = 0, 1, 2, and 3) HEAMCs, pure elemental powders related to Cr, Fe, Cu, Mn, and Ni, and ultra-fine α-Al_2_O_3_ (105 nm) were procured from Messrs (M/s) Nanografi, Jena, Germany. The purity of as-received powder was ensured to be more than 99.5%. The atomic and weight percentages of the synthesized HEAMCs are listed in Table 1. An electronic weighing balance with four digit accuracy was used to weigh the powders, as shown in Table 1, and charged into a four-station planetary high-energy ball mill (HEBM). M/s. TENCON, XQM-4A, Chin, HEBM was used and run in auto-mode according to the parameters. The set parameters during high-energy ball milling (milling media: hardened stainless-steel balls and vials) were 50 h of milling time, 15:1 ball-to-powder ratio, 350 rpm milling speed, 15 min forward, 15 min pause, and 15 min reverse. Ethanol was used as a process control agent (PCA) to minimize cold welding and oxidization during the MA process. The mechanically alloyed powders were dried and the stress was relieved inside a vacuum tube furnace at 393 ± 30 K (M/s. Nabatherm, Luxembourg, Germany).

### 2.2. Hot Compaction, Medium-Frequency Sintering, and Hot Forging

To prepare the bulk samples, stress-relieved powders were charged into the H13 die-set, heated to 823 ± 20 K, held for 0.75 h, and then, hot-compacted at 550 MPa in a universal testing machine (M/s MTS, USA: 250 kN capacity) and held for 10 min. An H13 steel die-set (heat-treated, 65 HRC) with a bore of 15 mm and a punch diameter of 15 mm was used [33]. A hot pellet with a diameter of 15 mm and a height of 25 ± 1 mm was produced by hot compaction. A graphite lubricant was applied to the die-wall surface before charging the powder. At least five pellets were prepared to study the mechanical behaviour, five samples with the same dimensions were produced to examine the wear behaviour, and three hot pellet samples with a height of 10 mm were used for X-ray diffraction and microstructural characterizations.

**Figure 1:**
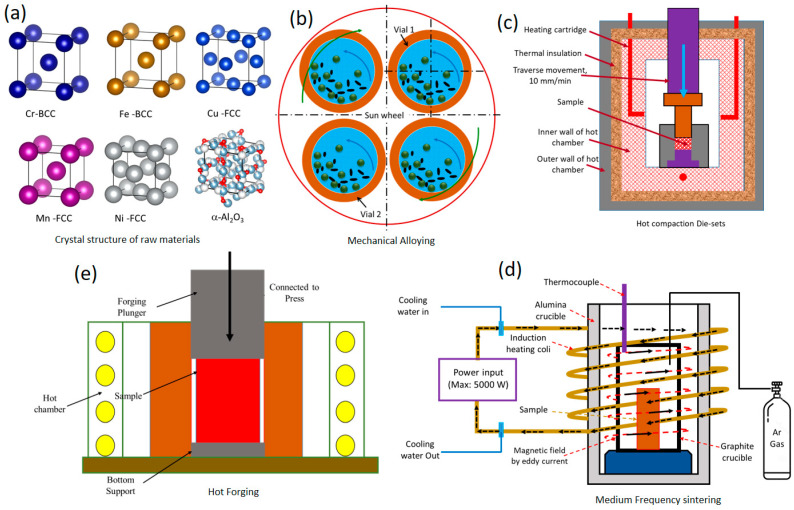
Schematic representing the development of CrFeCuMnNi-x vol.% *γ*-Al_2_O_3_ (0, 1, 2, and 3) HEAMCs: (**a**) crystal structure of the raw materials; (**b**) mechanical alloying process; (**c**) hot compaction; (**d**) medium-frequency sintering; and (**e**) hot forging.

The hot-compacted samples were then subjected to medium-frequency sintering in an argon atmosphere. A medium-frequency electric induction furnace manufactured by M/s Zhengzhou Yuanjie Chemical co., Ltd., Zhengzhou, China was used. The maximum heat input capacity of the furnace was 4000 W and 2000 W was used with a heating rate of 120°/min. Sintering was conducted at 1473 ± 20 K and held at this temperature for approximately 15 min; the sintered hot-pellets were transferred to a graphite-coated pre-heated die of 16 mm in diameter (H13 steel, preheated to 750 °C), and forged at 1273 ± 40 K with 50 MPa forging pressure (forging ratio: 1.13:1) and then air-cooled. Figure 1 shows a schematic diagram representing the unit cell of the as-received powders, MA process, hot compaction, medium-frequency sintering, and hot forging.

### 2.3. Characterization Using HRSEM, XRD, and EBSD

HRSEM (M/s Apreo FEG) was used to examine the surface morphologies of the as-received powder, milled powders, and forged samples. This HRSEM was operated at 30 keV with an accuracy of 1.3 nm resolution at 1 keV. An X-ray diffraction machine (M/s Empyrean, Malven Panalytical) was used to characterize the as-received, milled, and forged samples. The XRD test was performed at a scanning speed of 0.6°/min and step size of 0.01°. Various phase formations were analysed using the X’Pert high score plus software. HRSEM with EBSD (electron backscatter diffraction) detector was used to examine the detailed microstructural analyses in terms of the grain coloured map of inverse pole figures (IPF), grain size distribution, and misorientation angle distribution. For EBSD analyses, the hot-forged samples were polished with electropolishing in an electrolyte, which consisted of 80% methanol and 20% perchloric acid.

### 2.4. Density Measurement and Mechanical Testing (Hardness, Compression, and Wear Test) of HEAMCs

The density of the hot-forged CrFeCuMnNi-x vol.% Al_2_O_3_ HEAMC samples was determined by the Archimedes principle, as shown in Equation (1):

(1) $$\begin{array}{c} Density\;of\;forged\;sample,\;\rho_{act}\\ =Relative\;density\;\left(R\right)\times density\;of\;water\;\left(\rho_{water}\right) \end{array}$$

The relative density (*R*) was calculated using Equation (2):

(2) $$R=\frac{Mass\;of\;sample\;in\;air\;\left(m_{air}\right)}{\left[mass\;of\;sample\;in\;air\;(m_{air})-mass\;of\;sample\;in\;water\;(m_{water})\right]}$$

The percentage theoretical density (%TD) was determined by Equation (3):

(3) $$\%TD=\frac{Actual\;density\;(\rho_{act})}{Theoretical\;density\;(\rho_{th})}\times 100$$

where $\rho_{th}$ is calculated based on the rule of mixtures as per Equation (4):

(4) $$\rho_{th}=\sum_{i=1}^{n}f_{i}\rho_{i}$$

where $f_{i}$ is the volume fraction of the incorporated metallic elements and $\rho_{i}$ is the theoretical density of the added powders. At least five measurements were performed in each trial, and the average was used for interpretation. Mechanical testing was performed using the Vickers microhardness and compression tests. Before executing the Vickers hardness test, the forged bulk samples were ground using SiC papers of different grit sizes and polished using a ceramic lapping paste to ensure a scratch-free surface. A load (*F*) of 100 kgf/mm^2^ was applied and held for 15 s. The indentation size (*d*) was measured and, then, the Vickers hardness number (VHN) was determined using Equation (5) [34]:

(5) $$VHN=1.854\frac{F}{d^{2}}$$

Compression tests on the forged samples were performed using a 250 kN capacity universal testing machine made by M/s MTS Corporation, USA. The samples were prepared according to the ASTM E 9 standard (ϕ13 × 18 mm). The tests were performed at a loading rate of 1 mm/min. A data acquisition system with test-work software was used to capture the data. Applied compressive load (*P*) and compression (deformation, δ) were used to determine the mechanical properties. The engineering compressive stress ($\sigma_{c}$) was determined using Equation (6):

(6) $$\sigma_{c}=\frac{Compressive\;load\;\left(P\right)}{Initial\;cross\;sectional\;area\;\left(A\right)}$$

The engineering compressive strain (ε) was determined using Equation (7):

(7) $$\varepsilon_{c}=\frac{Change\;in\;deformation\;\left(\delta \right)}{Initial\;sample\;height\;\left(h\right)}$$

### 2.5. Sliding Wear Test

**Figure 2:**
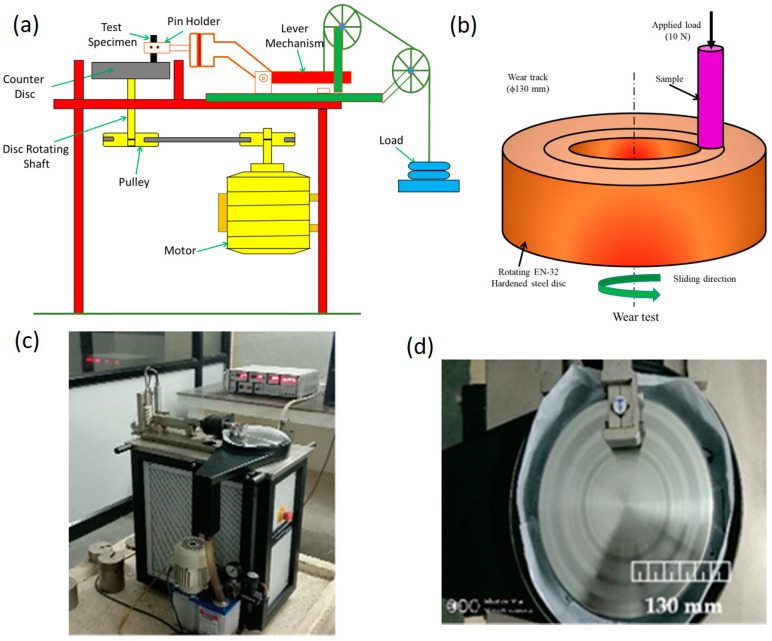
Schematic diagram representing: (**a**) wear test apparatus used in this work; (**b**) sliding wear principle by pin-on-disc approach; (**c**) photograph of the wear test apparatus used in this work; and (**d**) photograph of the pin and counter disc.

A tribometer (M/s DUCOM, Banglore, India) was used to conduct a sliding wear test on the developed HEAMCs at room temperature. Previously, the samples were prepared with 10 mm diameter and 16 mm height (by wire-cut EDM) and the test was conducted according to the ASTM G99 standard. The samples acted as pins in the sample holder, which was attached to an external load. A counter disc made of EN-32-hardened chromium steel was used. This counter disc has a Rockwell hardness of 63 HRC with 0.55 ± 2 μm surface roughness. A load of 10 N, sliding distance of 2000 m, sliding speed of 3 m/s, track distance of 130 mm, and sliding time of 11.11 min were used during the wear tests [35,36,37,38]. A load cell was used to measure the induced frictional force (F) and a linear variable differential transducer was used to measure the log wear depth. Five trials were used during the wear test and the average was used for the investigation. Mass loss was calculated before and after the wear tests. Figure 2 shows a schematic of the wear test apparatus used in this study (Figure 2a) and the sliding wear test using the pin-on-disc approach (Figure 2b). Figure 2c,d shows photographs of the sliding wear test apparatus used in this study. The wear performance was investigated using Equations (8)–(10):

(8) $$Specific\;wear\;rate,\;W_{R}=\frac{Worn\;surface\;volume\;\left(WSV\right)}{Applied\;load\;\left(P\right)\times Sliding\;distance\;\left(D\right)},\;\frac{m^{3}}{Nm}$$

(9) $$W_{R}=\frac{Mass\;loss\;\left(\Delta m\right)}{Applied\;load\;\left(P\right)\times Theoretical\;density\;\left(\rho \right)\times Sliding\;distance\;\left(D\right)},\;\frac{m^{3}}{Nm}$$

(10) $$Coefficient\;of\;friction\;\left(CoF\right)=\frac{Friction\;force\;\left(F\right)}{Thrust\;force\;\left(P\right)}$$

## 3. Results and Discussion

### 3.1. Examination of the Powder Surface Morphology of HEAMCs

**Figure 3:**
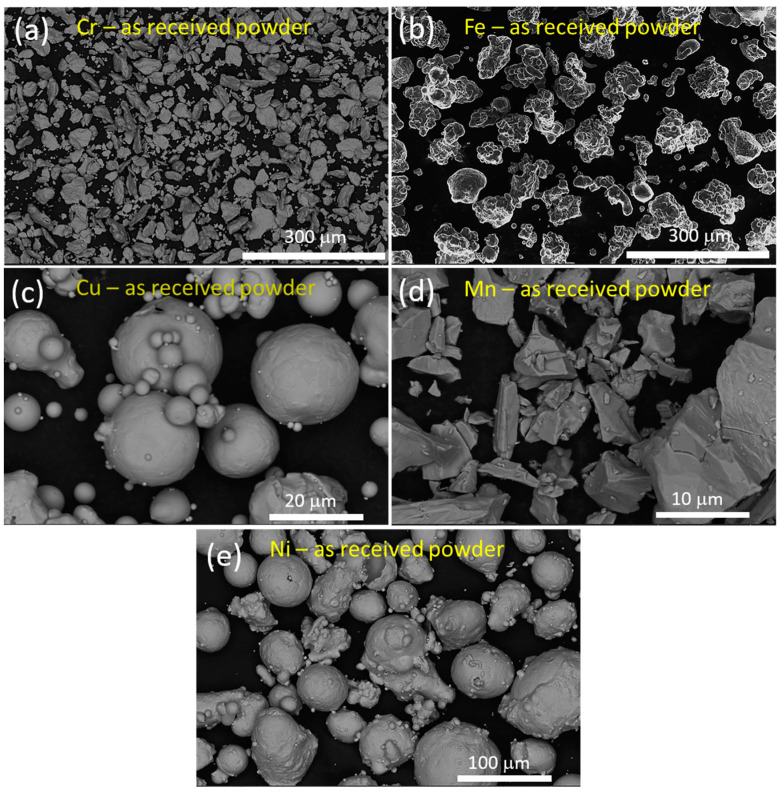
HRSEM images of the as-received elemental powders: (**a**) Cr; (**b**) Fe; (**c**) Cu; (**d**) Mn; and (**e**) Ni.

**Figure 4:**
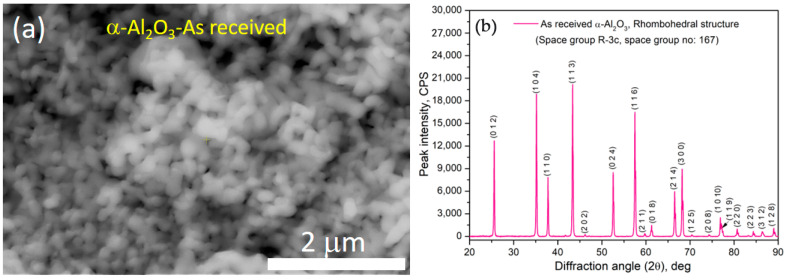
(**a**) HRSEM image of the as-received *α*-Al_2_O_3_ powders; (**b**) corresponding X-ray diffraction profile of *α*-Al_2_O_3_.

Figure 3 shows the HRSEM-backscattered electron (BSE) images of the as-received elemental powder surface morphology. It was observed that irregular flake-like shapes with an average particle size of 48 ± 3.4 μm (Figure 3a), irregular and equiaxed shapes with an average size of 45 ± 2.8 μm (Figure 3b), regular spherical shapes with an average size of 26 ± 6.45 μm (Figure 3c), irregular polygonal shapes with an average size of 27 ± 5.78 μm (Figure 3d), and spherical shapes attached to some satellite particles with an average size of 20.5 ± 3.89 μm (Figure 3e) were noticed for Cr, Fe, Cu, Mn, and Ni, respectively. The HRSEM BSE image of the as-received α-Al_2_O_3_ powder particles is shown in Figure 4a. From Figure 4a, it is clear that almost spherical particles in the agglomerated condition were observed in the as-received condition. Based on several BSE images and ImageJ software, the average particle size of the as-received α-Al_2_O_3_ was 105 ± 3.5 nm. The formation of agglomeration is attributed to van der Waals forces of attraction and a higher surface energy, which is a common phenomenon in nanoparticles [39]. The X-ray diffraction (XRD) peak profile of the as-received α-Al_2_O_3_ powder is shown in Figure 4b, in which the peaks related to Al_2_O_3_ at different planes were observed and the crystal structure of the as-received Al_2_O_3_ was rhombohedral (space group: R-3C, and space group no: 167). Figure 4b does not show any other unusual peaks, which confirms the purity of the as-received Al_2_O_3_ powders.

**Figure 5:**
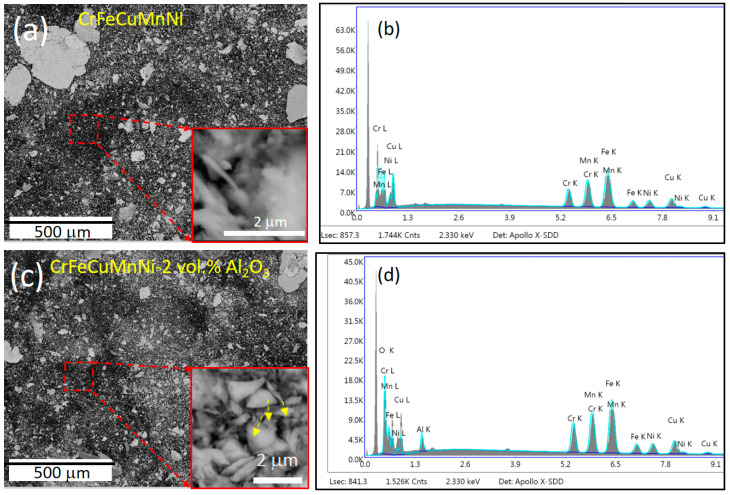
HRSEM powder surface morphologies and EDAX spectrum for the developed HEAMCs: (**a**) powder surface morphology of CrFeCuMnNi (inset shows the solid solution formation); (**b**) the corresponding EDS of (**a**); (**c**) powder surface morphology of CrFeCuMnNi-2 vol.% Al_2_O_3_ (inset shows the solid solution formation and dispersion of Al_2_O_3_ particles); and (**d**) the corresponding EDS of (**c**).

**Figure 6:**
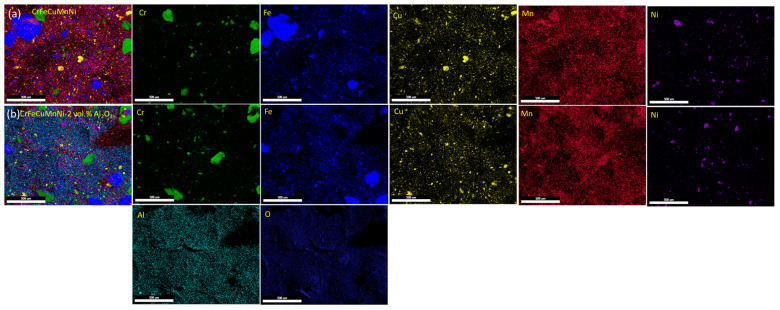
HRSEM elemental overlay map analyses on the developed high-entropy alloy matrix composites (HEAMCs): (**a**) CrFeCuMnNi; and (**b**) CrFeCuMnNi-2 vol.% Al_2_O_3_.

To confirm the solid solution formation and dispersion of the incorporated Al_2_O_3_ ceramic particles, HRSEM BSE images were captured for the CrFeCuMnNi alloy matrix (Figure 5a,b) and CrFeCuMnNi-2 vol.% Al_2_O_3_ HEAMC (Figure 5c,d) samples after 50 h of the MA process. Almost equiaxed powder particles were observed in both samples, indicating the attainment of a steady state [40]. Most of the powder particles were very fine in size, and a few particles exhibited a flake-like shape with a large size. More flake-like large powder particles were observed in the CrFeCuMnNi of the unreinforced alloy (Figure 5a), whereas fewer flake-like large powder particles were observed in the CrFeCuMnNi-2 vol.% Al_2_O_3_ HEAMCs sample (Figure 5b). Based on several images and using ImageJ software, the average sizes of the CrFeCuMnNi and CrFeCuMnNi-2 vol.% Al_2_O_3_ samples were 2.1 μm, and 1.1 μm, respectively. The formation of a very fine matrix (2.1 μm size of unreinforced HEA and 1.1 μm size of HEMCs) and composite particles was attributed to the high energy imported into charged materials by HEBM and the high-configurational entropy effect. These results demonstrate that the matrix powder particle size decreased by 47% in the CrFeCuMnNi-2 vol.% Al_2_O_3_ sample compared to that of the unreinforced sample. This was attributed to the incorporation of Al_2_O_3_ particles that acted as the milling medium. Repeated cold welding and fracturing are the main mechanisms involved in the MA process owing to the kinetic mechanical collisions produced by hardened stainless-steel balls and vials. Cold welding produces agglomerated large powder particles, whereas the fracturing mechanism helps to decrease the particle size. The insets at the bottom of Figure 5a,c show the attainment of a solid solution and the homogenization of the powder particles after 50 h of MA. The yellow arrow in the bottom inset of Figure 5c represents the dispersion of Al_2_O_3_ particles in the matrix powders, which confirms the successful production of HEAMCs. The HRSEM EDS spectra of both samples are shown in Figure 5b,c, which show all elements related to the incorporated one. The results confirm the composite nature of the milled powders as the presence of O and Al peaks in Figure 5d was observed in addition to Cr, Fe, Cu, Mn, and Ni peaks. Figure 6 shows the elemental overlay maps of the CrFeCuMnNi and CrFeCuMnNi-2 vol.% Al_2_O_3_ samples. These results also indicate the formation of a solid solution, homogeneous state, and dispersion of Al_2_O_3_ particles in the CrFeCuMnNi-2 vol.% Al_2_O_3_ sample (Figure 6b).

### 3.2. X-ray Diffraction Phase Analyses on HEAMCs

**Figure 7:**
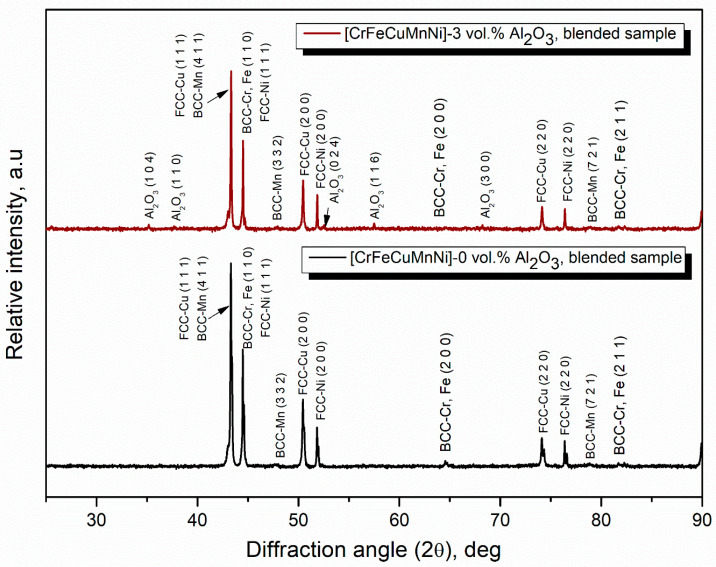
X-ray diffraction peak profile of the blended (0 h) samples of CrFeCuMnNi and CrFeCuMnNi-3 vol.% Al_2_O_3_ HEAMCs.

**Figure 8:**
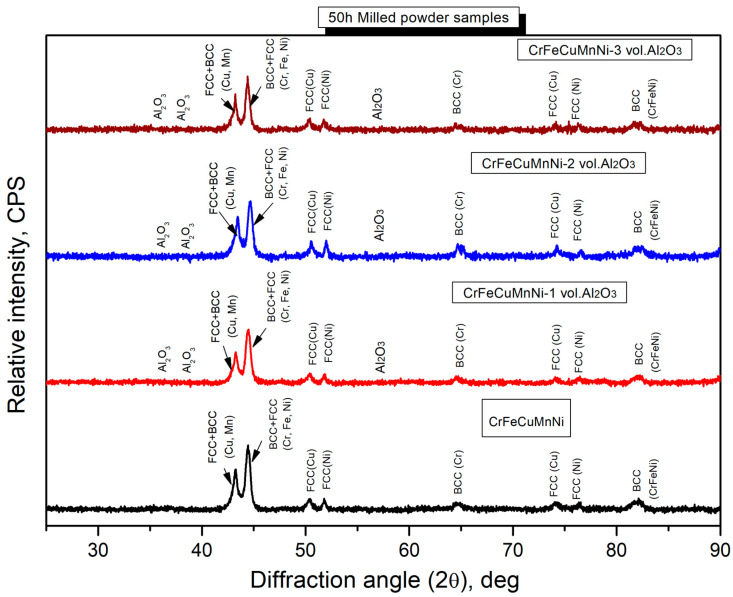
X-ray diffraction peak profile of the 50 h milled HEAMCs of CrFeCuMnNi, CrFeCuMnNi-1 vol.% Al_2_O_3_, CrFeCuMnNi-2 vol.% Al_2_O_3_, and CrFeCuMnNi-3 vol.% Al_2_O_3_.

Figure 7 shows the X-ray diffractometer results of the blended (0 h) samples of CrFeCuMnNi and CrFeCuMnNi-3 vol.% Al_2_O_3_, exhibiting high intensity with narrow peaks owing to the lack of kinetic energy given to the charged powders. The X-ray peak profiles of the 50 h mechanically alloyed powders of the CrFeCuMnNi, CrFeCuMnNi-1 vol.% Al_2_O_3_, CrFeCuMnNi-2 vol.% Al_2_O_3_, and CrFeCuMnNi-3 vol.% Al_2_O_3_ HEAMC samples are shown in Figure 8. The results demonstrate that the observed peak profiles decreased extensively and peak broadening was observed. In addition, several peaks vanished and merged into single peaks, indicating the formation of a solid solution. Major body-centred cubic (BCC), face-centred cubic (FCC), and Al_2_O_3_ phases were observed, which confirms the successful fabrication of HEAMCs. Table 2 lists the atomic radii, crystal structures, and physical properties of the incorporated metallic elements and the ceramic particles. As expected, a supersaturated solid solution (Figure 5 and Figure 6) was easily obtained because the atomic radii of the incorporated elements were similar (Table 2). A severe reduction in peak intensity and a drastic increase in peak broadening were expected owing to the 50 h MA process, which introduced the kinetic energy of mechanical collisions obtained from the milling medium (stainless-steel balls and vials) [40].

**Figure 9:**
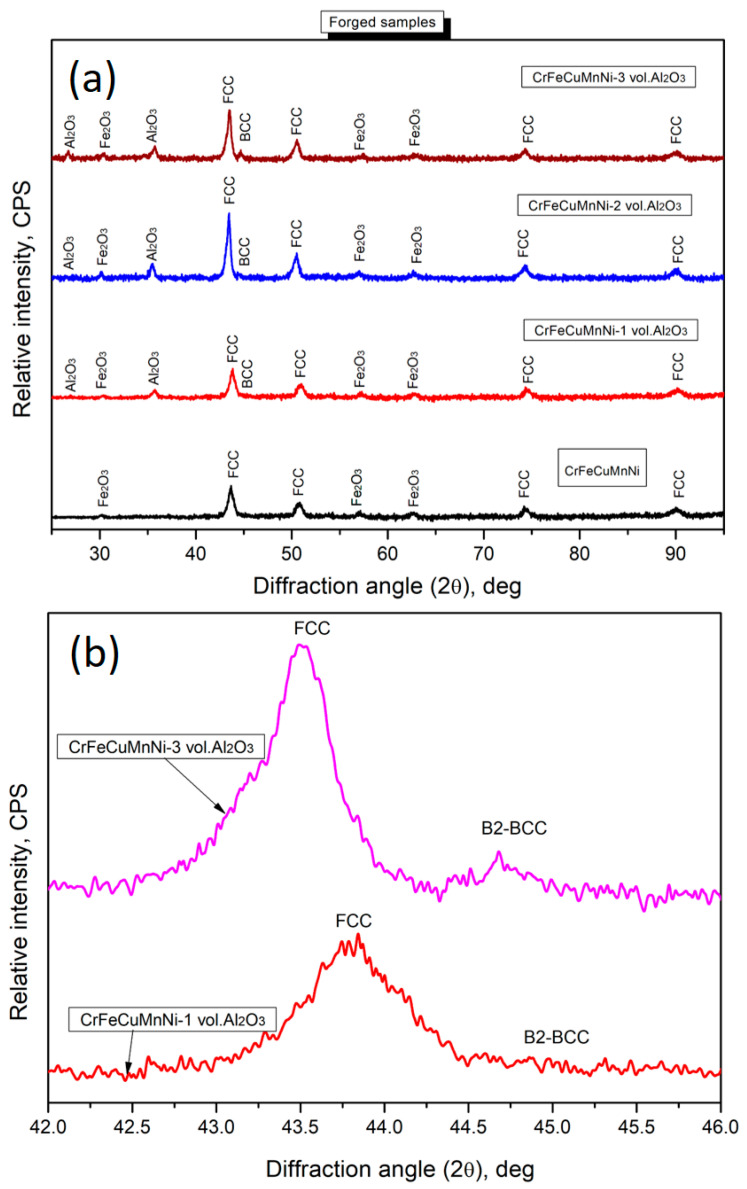
X-ray diffraction peak profile of the hot-forged HEAMCs of: (**a**) CrFeCuMnNi, CrFeCuMnNi-1 vol.% Al_2_O_3_, CrFeCuMnNi-2 vol.% Al_2_O_3_, and CrFeCuMnNi-3 vol.% Al_2_O_3._, (**b**) magnified view (**a**) showing the peak shift.

The X-ray diffractometer results of the hot-forged CrFeCuMnNi, CrFeCuMnNi-1 vol.% Al_2_O_3_, CrFeCuMnNi-2 vol.% Al_2_O_3_, and CrFeCuMnNi-3 vol.% Al_2_O_3_ HEAMCs are shown in Figure 9a, showing a major FCC phase and a minor B2-BCC phase. The major BCC phase observed in the 50 h milled powders almost disappeared after hot forging due to high-temperature sintering followed by hot forging and air cooling. Some authors [41,42] also observed the disappearance of the major BCC phase and the appearance of a major FCC phase after consolidation. Figure 9b shows the magnified view representing the major peak shift occurs with the variation of Al_2_O_3_ content due to more structural refinement. Further, Table 3 list the calculated internal structural parameters of hot-forged samples. The presence of a major FCC and minor B2-BCC phases along with the incorporated Al_2_O_3_ particles was expected to improve the mechanical and wear properties of the fabricated HEAMCs [43,44]. In addition, the Fe_2_O_3_ phase was observed in all hot-forged HEAMCs samples owing to contamination from the milling medium and high-temperature sintering followed by forging and air cooling.

### 3.3. Physicochemical and Thermodynamic Parameters of the Developed HEMCs

Various parameters, namely, Gibbs free energy (ΔG_mix_), enthalpy of mixing (ΔH_mix_), entropy of mixing (ΔS_mix_), thermodynamic parameter (Ω), atomic size deviation (*δ*), valence electron concentration (VEC), and electro-negativity (ΔX), can be used for predicting the phase formation and the phase stability of the developed HEAMCs. All parameters were calculated based on Equations (11)–(19) [45,46].

ΔG_mix_ = ΔH_mix_ − TΔS_mix_ (11)

(12) $${\Delta S}_{\mathrm{mix}}=-R\sum_{i=1}^{N}(C_{i}lnC_{i})$$

where R is the gas constant (8.314 J/mol·K) and *C_i_* represents the atomic fraction of the *i*th element.

(13) $${\Delta H}_{\mathrm{mix}}=\sum_{i=1,\;i\neq j}^{N}(4{\Delta H}_{mix}^{AB}C_{i}C_{j})$$

where ${\Delta H}_{mix}^{AB}$ is the mixing enthalpy between the *i*th and *j*th elements (Table 4 [47]).

(14) $$\Omega =\frac{T_{m}{\Delta S}_{\mathrm{mix}}}{{\Delta H}_{\mathrm{mix}}}$$

where *T_m_* is the melting point in K (see Table 2).

(15) $$\delta =\sqrt{\sum_{i=1}^{N}C_{i}{\left(1-\frac{r_{i}}{\bar{r}}\right)}^{2}}$$

(16) $$\bar{r}=\sum_{i=1}^{N}C_{i}r_{i}$$

where *r_i_* is the atomic radius in m (see Table 2).

(17) $$\mathrm{VEC}=\sum_{i=1}^{N}C_{i}{\left(VEC\right)}_{i}$$

(18) $$\Delta X=\sqrt{\sum_{i=1}^{N}C_{i}{\left(X_{i}-\bar{X}\right)}^{2}}$$

(19) $$\bar{X}=\sum_{i=1}^{N}C_{i}X_{i}$$

where *X_i_* represents the Pauling electronegativity of the *i*th element.

The values of δH_mix,_ δS_mix,_ δG_mix,_ Ω, δ, VEC, and δX for the developed HEAMCs are listed in Table 5. The criteria for the formation of a solid solution were Ω > 1.1 and δ < 6.6%. From Table 5, it is clear that a solid solution was possible in the developed HEAMCs. Furthermore, the mixing entropy increased with the addition of Al_2_O_3_ and decreased with the formation of intermetallic phases after hot forging at elevated temperatures. VEC can be used to predict the phases and phase stabilities of the developed HEAMCs. The conditions for phase formation were: stable FCC phase formation when VEC > 8; single BCC phase formation when VEC < 6.87; and the mixing of FCC and BCC phase formation when 6.87 < VEC < 8 [48]. Based on the VEC values, the developed HEAMCs exhibited values greater than 8, indicating that the stable FCC phase was expected. The predicted phases matched the XRD results of the hot-forged samples (Figure 9). The minor B2-BCC phase formation was attributed to the Cr- and Fe-rich precipitates formed after the high-temperature forging process. The formation of the minor B2-BCC phase was attributed to the increase in the Pauling electronegativity difference (Table 5) with increasing the Al_2_O_3_ content in the CrFeCuMnNi HEA matrix [49]. Elements possessing high electronegativity differences are prone to losing or gaining electrons, which is expected to promote the formation of the B2-BCC phase in the developed HEAMCs.

### 3.4. HRSEM Microstructural Examination with EBSD Analyses on Hot-Forged HEAMCs

**Figure 10:**
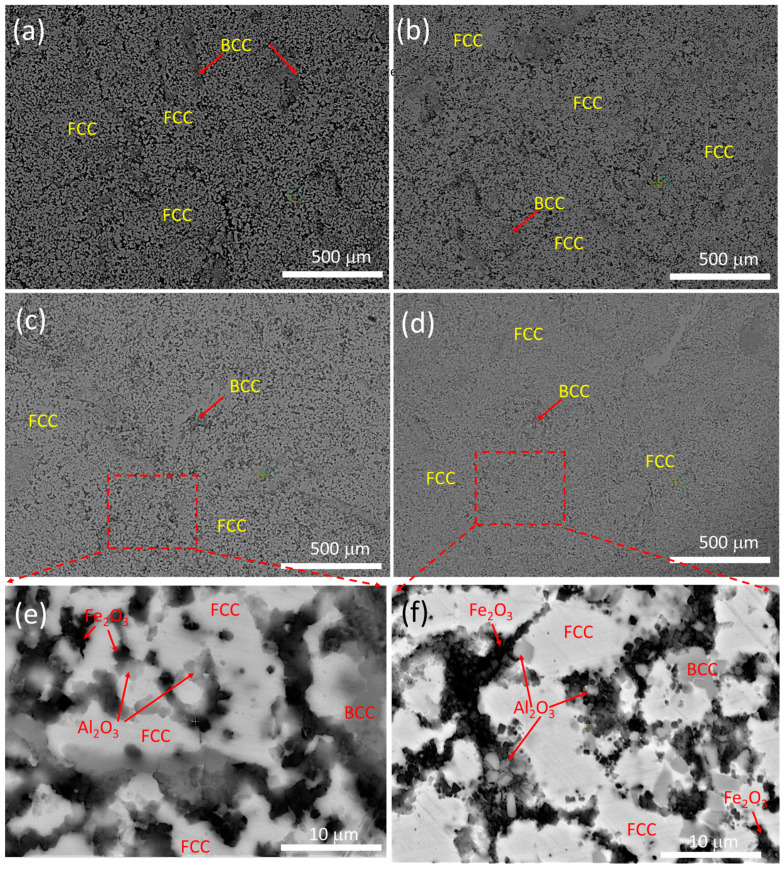
HRSEM microstructures of the hot-forged HEAMCs of: (**a**) CrFeCuMnNi; (**b**) CrFeCuMnNi-1 vol.% Al_2_O_3_; (**c**) CrFeCuMnNi-2 vol.% Al_2_O_3_; and (**d**) CrFeCuMnNi-3 vol.% Al_2_O_3_. (**e**,**f**) shows the magnified view of (**c**,**d**), respectively illustrating the dispersion of Al_2_O_3_ over matrix.

Figure 10a–d shows the HRSEM microstructures of the hot-forged HEAMCs that exhibited both the FCC phase (large area) and a small amount of the B2-BCC phase (dark region in a small area). These results correlate well with the XRD peak profiles of the hot-forged samples (Figure 9). It can be seen that the observed major FCC grains and minor BCC grains were refined with the addition of Al_2_O_3_, indicating that the incorporated Al_2_O_3_ particles decreased the CrFeCuMnNi HEA matrix grain growth. Figure 10e,f show the magnified HRSEM microstructures of the CrFeCuMnNi-2 vol.% Al_2_O_3_ and CrFeCuMnNi-3 vol.% Al_2_O_3_ samples, respectively. The dispersion of the incorporated Al_2_O_3_ particles was observed in both samples; however, the little agglomeration of the Al_2_O_3_ particles over the HEA matrix grain boundaries was observed in the CrFeCuMnNi-3 vol.% Al_2_O_3_, indicating the limit of the addition of Al_2_O_3_ particles in the developed HEAMCs. Furthermore, the formation of the partial dissolution and overlapping of the B2-BCC phase over the FCC phase was observed because of the hot-forging process; consequently, improved mechanical properties were expected.

**Figure 11:**
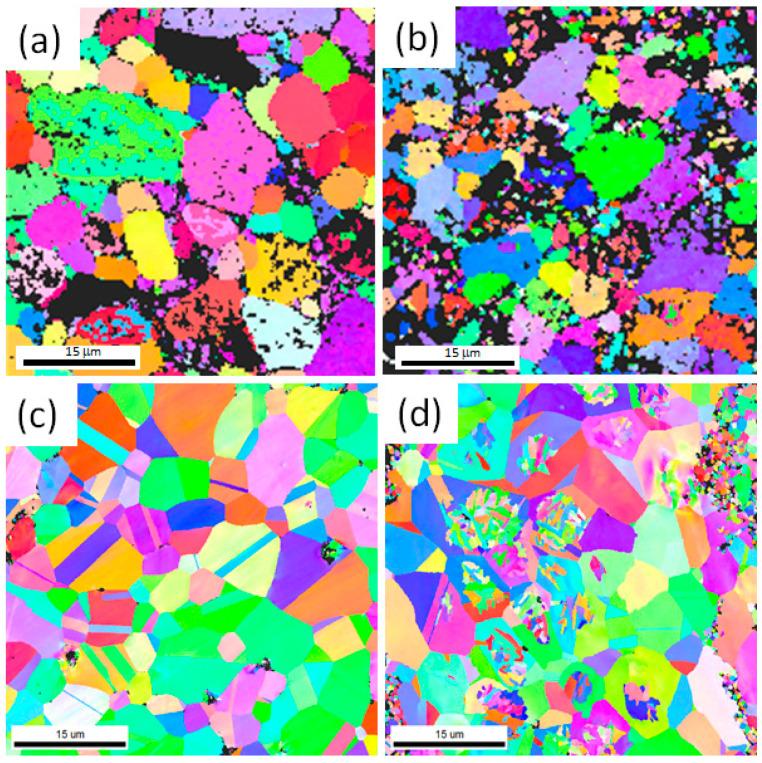
HRSEM with EBSD inverse pole figures (IPFs) of the developed HEAMCs of: (**a**) CrFeCuMnNi; (**b**) CrFeCuMnNi-1 vol.%Al_2_O_3_; (**c**) CrFeCuMnNi-2 vol.% Al_2_O_3_; and (**d**) CrFeCuMnNi-3 vol.% Al_2_O_3_.

**Figure 12:**
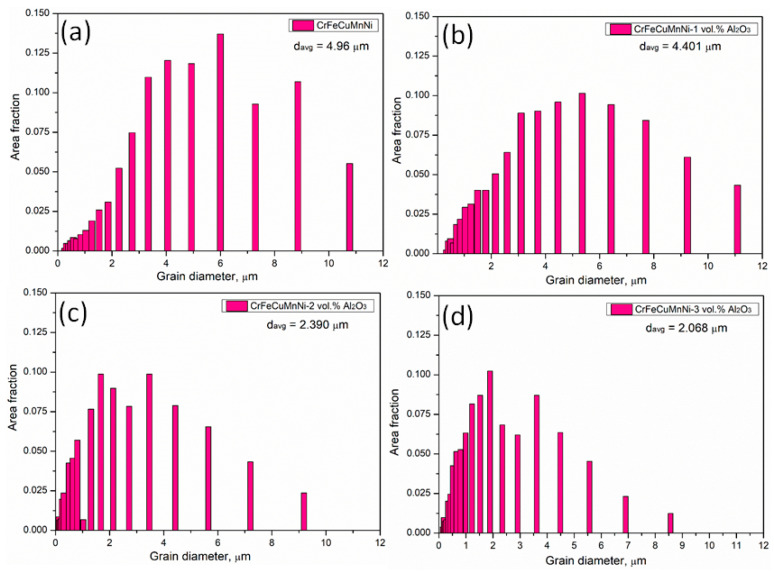
CrFeCuMnNi matrix grain size distribution of the developed HEAMCs over the area fraction: (**a**) CrFeCuMnNi alloy; (**b**) CrFeCuMnNi-1 vol.% Al_2_O_3_; (**c**) CrFeCuMnNi-2 vol.% Al_2_O_3_; and (**d**) CrFeCuMnNi-3 vol.% Al_2_O_3_.

**Figure 13:**
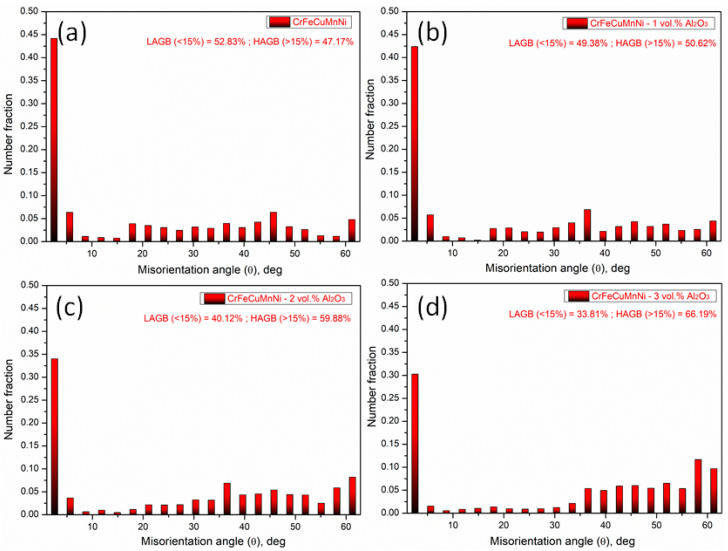
Misorientation angle distribution over the number fraction of the developed HEAMCs of: (**a**) CrFeCuMnNi alloy; (**b**) CrFeCuMnNi-1 vol.% Al_2_O_3_; (**c**) CrFeCuMnNi-2 vol.% Al_2_O_3_; (**d**) and CrFeCuMnNi-3 vol.% Al_2_O_3_.

HRSEM with EBSD analyses was conducted for the developed HEAMCs to examine the grain size of the matrix by inverse pole figures (IPFs) and grain refinements by grain size and misorientation angle distribution. An FEI Quanta FEG SEM with TSL-OIM software was used to examine the EBSD data and the linear intercept method was used to measure the grain size. Figure 11 shows the EBSD inverse pole figures (IPFs) of the developed HEAMCs, which clearly demonstrate the variation in the CrFeCuMnNi matrix grain size with the addition of Al_2_O_3_ particles. The observed grain size started to decrease with the increase in Al_2_O_3_ particles owing to the solid solution formation, dislocation pinning by Al_2_O_3_ particles, high-configurational entropy, and structural refinement of MA and hot-forging processes. In addition, the observed matrix grain size was almost equiaxed in shape, indicating an isotropic nature [50]. The grain size distribution of the CrFeCuMnNi matrix based on the EBSD microstructures over the area fraction was determined and is shown in Figure 12. The results demonstrate that the CrFeCuMnNi matrix grain size decreased with the increase in Al_2_O_3_ particles, confirming the grain refinement process by MA followed by the hot-forging process [51]. The average matrix grain sizes were 4.96, 4.40, 2.39, and 2.07 μm for CrFeCuMnNi, CrFeCuMnNi-1 vol.% Al_2_O_3_, CrFeCuMnNi-2 vol.% Al_2_O_3_, and CrFeCuMnNi-3 vol.% Al_2_O_3_, respectively. The misorientation angle distribution over the number fraction of developed HEAMCs is shown in Figure 13. The misorientation angle of the observed grains represents structural refinement. Low-angle grain boundaries (LAGBs, <15°) indicate a coarse grain nature, whereas high-angle grain boundaries (HAGBs, >15°) denote the fine-grained nature of the samples. In this study, the observed percentages of LAGBs and HAGBs were 52.83% and 47.17%, 49.38% and 50.62%, 40.12% and 59.88%, and 33.81% and 66.19% for CrFeCuMnNi, CrFeCuMnNi-1 vol.% Al_2_O_3_, CrFeCuMnNi-2 vol.% Al_2_O_3_, and CrFeCuMnNi- 3 vol.% Al_2_O_3_, respectively. The results indicate that the value of HAGBs started to increase with the increase in Al_2_O_3_ particles, ensuring an increase in structural refinement owing to the high-configurational mixing entropy effect, solid solution formation, dislocation pinning, and hot-forging process [50,52].

### 3.5. Examination of the Relative Density and Vickers Hardness of the Developed HEAMCs

**Figure 14:**
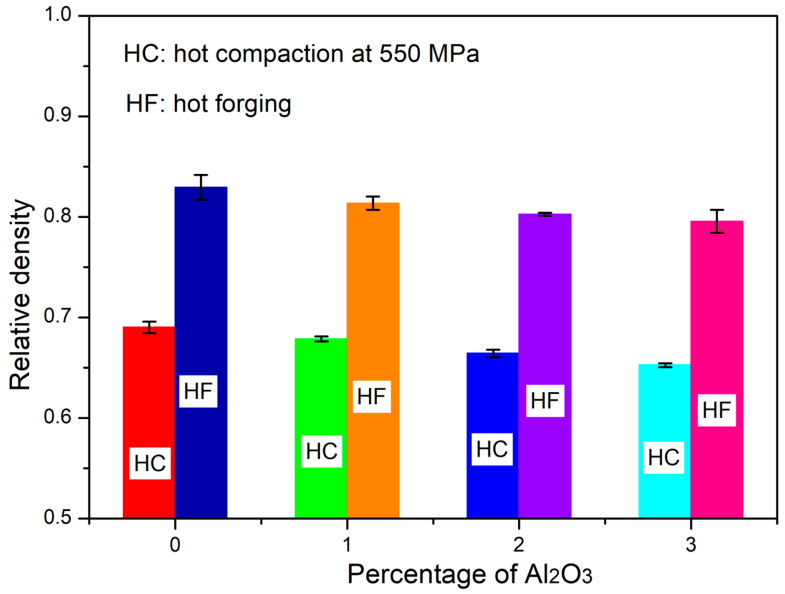
Variation of the relative density of the developed HEAMCs.

The relative density of the hot-compacted pellets and hot-forged samples were measured using Archimedes’ principle and the values are listed in Table 6. The variation in relative density with the volume percentage of Al_2_O_3_ in the CrFeCuMnNi HEA matrix is shown in Figure 14. The results show that the measured relative density decreased slightly with the increase in the amount of Al_2_O_3_ in the CrFeCiMnNi HEA matrix of both hot-compacted and hot-forged samples owing to the dislocation pinning of the Al_2_O_3_ particles over the grain boundaries (Figure 10e,f), increasing the structural refinement (Figure 10, Figure 11, Figure 12 and Figure 13), and a high-configurational mixing entropy effect (Table 5). The presence of fine Al_2_O_3_ particles over the grain boundaries, more structural refinement, and high-configurational entropy effect led to an increased resistance to packing during hot compaction and decreased dislocation motion during hot forging, which resulted in a decrease in the relative density. However, the relative density of the hot-forged samples were higher than those of the hot-compacted pellets. This was attributed to the decrease in the yield strength of the bulk samples during the high-temperature forging process, which led to a decrease in the height of the samples and consequently increased the density. For example, the relative density of the unreinforced CrFeCuMnNi matrix after hot compaction and hot forging were 0.6902 ± 0.0057 and 0.8295 ± 0.0124, respectively. The relative density of the hot-forged CrFeCuMnNi HEA matrix was 20.18% higher than that obtained under hot-compacted conditions. Similarly, the relative density of the CrFeCuMnNi-3 vol.% Al_2_O_3_ HEAMC sample exhibited 0.6525 ± 0.0019 and 0.7957 ± 0.0115 after hot compaction and hot forging, respectively. An increase of almost 21.95% in density was achieved in the hot-forged CrFeCuMnNi-3 vol.% Al_2_O_3_ HEAMC sample compared to hot-compacted one. This is attributed to grain growth during the high-temperature medium-frequency sintering followed by hot forging and a decrease in the yield strength at elevated temperatures. These results clearly demonstrate that the hot-forging process helps to enhance the relative density, which is a low-cost and easily applicable process in industries.

**Figure 15:**
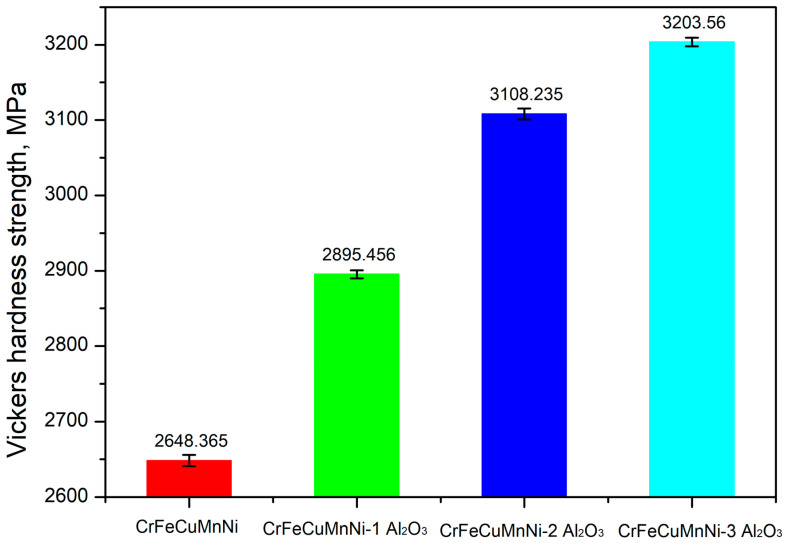
Variation of the Vickers hardness strength for the developed HEAMCs.

Figure 15 shows the increase in the Vickers hardness strength (VHS) as a function of Al_2_O_3_ in the CrFeCuMnNi HEA matrix. The VHS of the CrFeCuMnNi, CrFeCuMnNi-1 vol.% Al_2_O_3_, CrFeCuMnNi-2 vol.% Al_2_O_3_, and CrFeCuMnNi-3 vol.% Al_2_O_3_ HEAMC samples were 2.648, 2.895, 3.108, and 3.203 GPa, respectively. The Vickers hardness strength of the CrFeCuMnNi-3 vol.% Al_2_O_3_ HEAMC sample was 1.21 times higher than that of the unreinforced CrFeCuMnNi HEA matrix. The increase in the hardness with the Al_2_O_3_ addition was attributed to the dislocation pinning of the Al_2_O_3_ particles over the grain boundaries (Figure 10e,f), increasing the structural refinement (Figure 10, Figure 11, Figure 12 and Figure 13) and high-configurational mixing entropy effect (Table 5). Based on the XRD results in Figure 9 and HRSEM micrographs in Figure 10, it is clear that the formation of the B2-BCC phase increased with the addition of Al_2_O_3_ and the hardness of the B2-BCC phase was expected to be higher than that of the FCC phase owing to the refined B2-BCC aggregates (dark region in Figure 10). Therefore, the applied load during the Vickers test was expected to be absorbed by the incorporated Al_2_O_3_ particles followed by the FCC phase of the CrFeCuMnNi matrix. The same observation was reported during the hot forging of the AlxCoCrFeNi alloys [50,53].

### 3.6. Mechanical Performance of the HEAMCs

**Figure 16:**
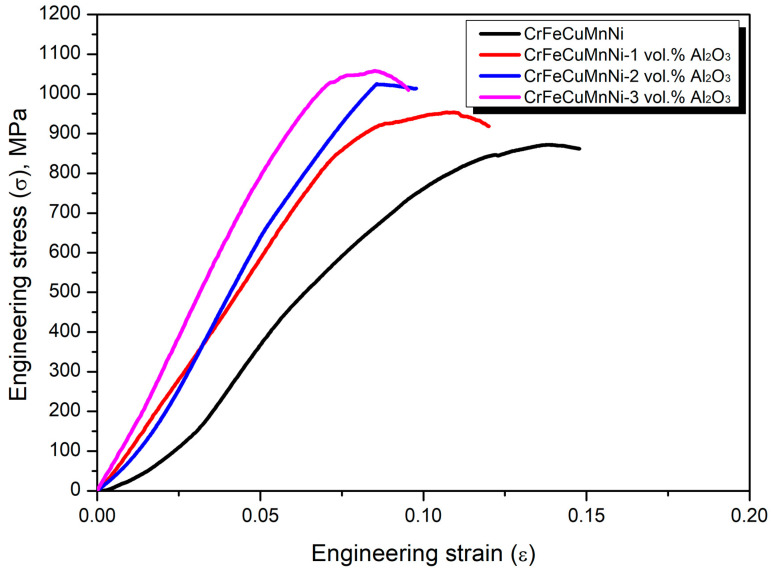
Compressive engineering stress–strain curves of the hot-forged CrFeCuMnNi-x vol.% Al_2_O_3_ HEAMCs.

**Figure 17:**
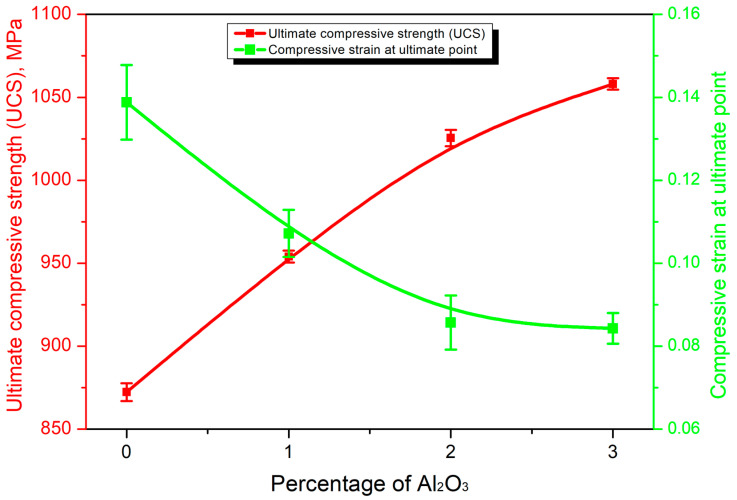
Variation of the ultimate compressive strength (UCS) and compressive strain at the ultimate point with the function of Al_2_O_3_ in CrFeCuMnNi HEAMCs.

**Figure 18:**
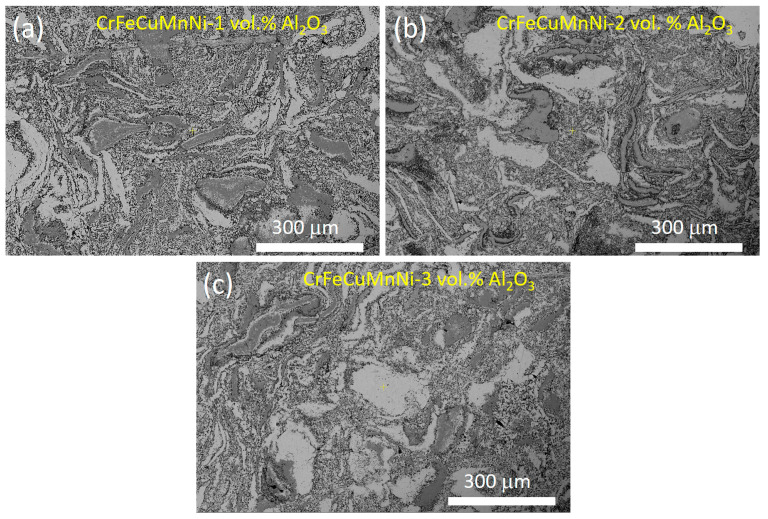
HRSEM microstructures of the deformed samples after the compression test of: (**a**) CrFeCuMnNi-1 vol.% Al_2_O_3_; (**b**) CrFeCuMnNi-2 vol.% Al_2_O_3_; and (**c**) CrFeCuMnNi-3 vol.% Al_2_O_3_ (microstructures captured at the outer periphery of the sample, which is perpendicular to the loading direction).

Figure 16 shows the compressive engineering stress–strain curves of the CrFeCuMnNi-x vol.% Al_2_O_3_ HEAMCs. The ultimate compressive strength (UCS) and the corresponding compressive strain at the ultimate point are shown in Figure 17. The results of Figure 16 and Figure 17 demonstrate that the compressive strength of the developed HEAMCs increased steadily with the increase in Al_2_O_3_ in the CrFeCuMnNi matrix. However, the corresponding strain at the ultimate point decreased, indicating a decrease in plasticity. The microstructure development after hot foring decreased the grain size of the CrFeCuMnNi matrix (Figure 10, Figure 11 and Figure 12). In general, grain refinement can enhance the strength and suppress ductility, owing to the formation of more grain boundaries (producing more surface defects) and leading to a diminished dislocation motion [54]. The UCS values of 872, 954, 1025, and 1058 MPa were obtained for the CrFeCuMnNi, CrFeCuMnNi-1 vol.% Al_2_O_3_, CrFeCuMnNi-2 vol.% Al_2_O_3_, and CrFeCuMnNi-3 vol.% Al_2_O_3_, respectively. CrFeCuMnNi-3 vol.% Al_2_O_3_ exhibited the highest UCS, which was around 1.21% times higher than that of the unreinforced CrFeCuMnNi matrix. The increase in the UCS with Al_2_O_3_ in the CrFeCuMnNi HEA matrix is attributed to the embedding of Al_2_O_3_ particles (Figure 10e,f), dislocation pinning produced by Al_2_O_3_ particles (Figure 10e,f), solid solution of incorporated metallic elements (Figure 10), structural refinement (Figure 10), and high-configurational mixing entropy effect (Table 5). However, the strain at the ultimate point was 0.1388, 0.1072, 0.0857, and 0.0843 for the CrFeCuMnNi, CrFeCuMnNi-1 vol.% Al_2_O_3_, CrFeCuMnNi-2 vol.% Al_2_O_3_, and CrFeCuMnNi-3 vol.% Al_2_O_3_ samples, respectively, which decreased drastically (Figure 17). The strain at the ultimate point of the unreinforced CrFeCuMnNi HEA was 1.64 times higher than that of the CrFeCuMnNi-3 vol.% Al_2_O_3_ HEAMC sample. The decrease in strain as a function of Al_2_O_3_ in the CrFeCuMnNi matrix was attributed to the increase in resistance against the dislocation motion produced by the Al_2_O_3_ particles, the B2-BCC phase, structural refinement, and high-configurational entropy [55,56]. Figure 18 shows the HRSEM microstructures of the deformed samples observed perpendicularly to the loading direction near the circumference of the sample. The results clearly show that more deformed grains in the FCC phase were observed in the CrFeCuMnNi-1 vol.% Al_2_O_3_ sample (Figure 18a) in comparison with those of the CrFeCuMnNi-3 vol.% Al_2_O_3_ sample (Figure 18c). It can be concluded that the developed HEAMCs can be recommended for use in industrial applications, particularly in aerospace, defence, and automotive industries.

### 3.7. Examination of the Wear Behavior of the HEAMCs

**Figure 19:**
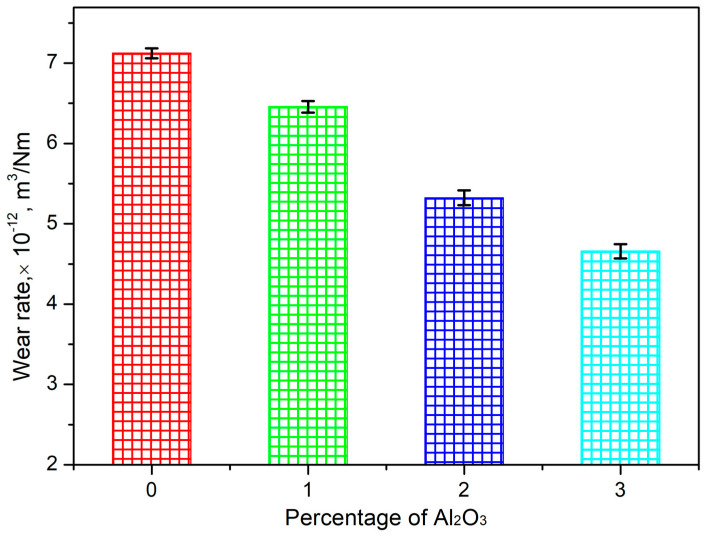
Variation in the wear rate (WR) with Al_2_O_3_ content in the CrFeCuMnNi of the hot-forged samples HEAMCs.

**Figure 20:**
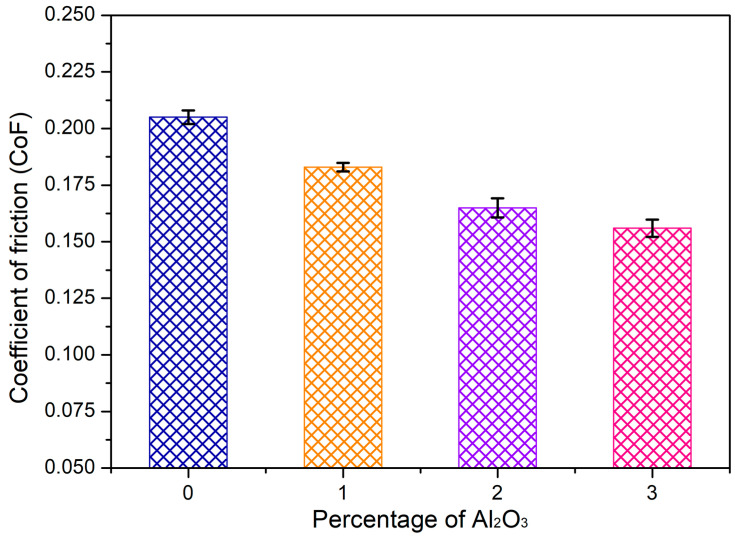
Variation in the coefficient of friction (CoF) with Al_2_O_3_ content in the CrFeCuMnNi of the hot-forged samples HEAMCs.

The influence of the Al_2_O_3_ content in the CrFeCuMnNi HEA matrix on the wear rate (WR) and coefficient of friction (CoF) of the developed hot-forged HEAMCs is illustrated in Figure 19 and Figure 20, respectively. The dry sliding wear rate at room temperature demonstrates that the wear rate decreased significantly with the increase in the content of Al_2_O_3_ in the CrFeCuMnNi HEA matrix. This is attributed to the solid solution of the incorporated metallic elements (Figure 6 and Figure 9), the effective dispersion of the Al_2_O_3_ particles in the matrix (Figure 10), structural refinement (Figure 11, Figure 12 and Figure 13), and high-configurational mixing entropy (Table 5). The WR of 7.12 × 10^−12^, 6.46 × 10^−12^, 5.32 × 10^−12^, and 4.66 × 10^−12^ m^3^/Nm for CrFeCuMnNi, CrFeCuMnNi-1 vol.% Al_2_O_3_, CrFeCuMnNi-2 vol.% Al_2_O_3_, and CrFeCuMnNi-3 vol.% Al_2_O_3_, respectively. The CrFeCuMnNi-3 vol.% Al_2_O_3_ HEAMC sample exhibited a 52.9% lower wear rate compared to the CrFeCuMnNi matrix. In addition, the increasing amount of the minor B2-BCC phase might have diminished the wear rate owing to the refined B2-BCC aggregates (dark region in Figure 10). These results clearly indicate that the wear resistance of the developed HEAMCs can be improved by the addition of Al_2_O_3_ particles, which suggests their potential for industrial applications. The experimental CoF value started to decrease considerably with the addition of Al_2_O_3_ to the CrFeCuMnNi matrix, indicating a decrease in friction, which can enhance the wear resistance of the developed HEAC samples. CoF values of 0.205, 0.183, 0.165, and 0.156 were observed for CrFeCuMnNi, CrFeCuMnNi-1 vol.% Al_2_O_3_, CrFeCuMnNi-2 vol.% Al_2_O_3_, and CrFeCuMnNi-3 vol.% Al_2_O_3_, respectively. The CrFeCuMnNi-3 vol.% Al_2_O_3_ HEAMC sample had around 31.41% lower CoF value compared to the CrFeCuMnNi matrix. The incorporation of Al_2_O_3_ particles and the formed Fe_2_O_3_ might have acted as a protective layer, consequently decreasing the metal contact between the sample (pin) and counter disc (hardened one), which leads to a decrease in CoF.

**Figure 21:**
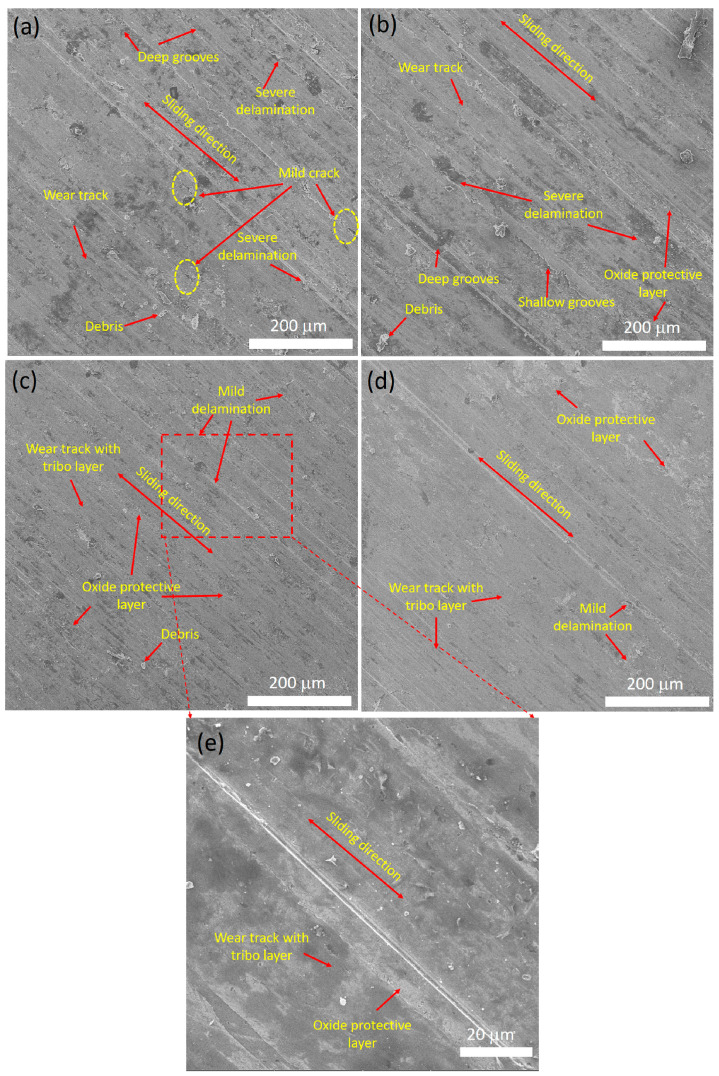
SEM worn surface morphology of the developed hot-forged samples after the dry sliding wear test of: (**a**) CrFeCuMnNi; (**b**) CrFeCuMnNi-1 vol.% Al_2_O_3_; (**c**) CrFeCuMnNi-2 vol.% Al_2_O_3_; (**d**) CrFeCuMnNi-3 vol.% Al_2_O_3_; and (**e**) magnified view of (**c**).

Figure 21 shows the SEM worn surface morphologies of the hot-forged samples after the sliding wear test at room temperature. It is clear that the incorporation of Al_2_O_3_ in the CrFeCuMnNi HEA matrix significantly influenced the wear performance. Deep grooves (more ploughing), severe delamination, and mild cracks were observed in the CrFeCuMnNi matrix (Figure 21a). In addition, more oxides on the outer surface of the wear track were formed in this sample, which clearly explains that the CrFeCuMnNi HEA matrix possesses a poor wear resistance owing to the domination of more abrasive and adhesive wear mechanisms [57]. This was attributed to a lower structural refinement (Figure 11a, i.e., large grain size), the absence of Al_2_O_3_ content (Figure 9), lower hardness (Table 6, Figure 15), and lower mechanical strength (Table 6 and Figure 17). However, with the increase in the content of Al_2_O_3_ in the CrFeCuMnNi matrix, the formation of deep grooves caused by ploughing, severe delamination, amount of debris, and the presence of oxides on the outer surface of the wear track decreased (Figure 21b–d). In addition, the formation of oxide particles inside the wear track, leading to the formation of an oxide protective layer, increased with the increase in the Al_2_O_3_ content in the CrFeCuMnNi matrix. These results demonstrate that the wear resistance increased with the addition of Al_2_O_3_ particles. In other words, the dominance of the adhesive and abrasive wear mechanisms decreased. A very smooth surface along with a tribo-layer was observed in the CrFeCuMnNi-3 vol.% Al_2_O_3_ sample (Figure 21d), indicating a high wear resistance compared to the other samples. A high-magnification SEM image of the worn surface morphology of CrFeCuMnNi-2 vol.% Al_2_O_3_ as an example is shown in Figure 21e, which shows the smooth surface and wear track with tribo-layer and oxide protective layer formation. The high wear resistance due to the presence of a smooth surface and wear track with a tribo-layer was attributed to the high-configurational mixing entropy effect (Table 5), the effective dispersion of Al_2_O_3_ particles over the matrix, structural refinement (Figure 10), and high mechanical strength (both compressive strength and hardness, Table 6 [49]). Overall, the addition of Al_2_O_3_ particles enhanced the wear resistance of the CrFeCuMnNi HEA matrix, which is suitable for both structural and defensive applications.

## 4. Conclusions

The major findings of this study are presented below:

❖ The developed hot-forged HEMCs exhibited a major face-centred cubic (FCC) structure that matched the predicted structure using physio- and thermo-mechanical parameters (ΔG_mix_, ΔH_mix_, ΔS_mix_, Ω, VEC, and ΔX) and a minor body-centred cubic (B2-BCC) phase owing to the high electronegativity difference and high temperature used during the hot-forging process.
❖ The addition of Al_2_O_3_ to the CrFeCuMnNi matrix enhances the high-configurational mixing entropy effect and decreases the mixing enthalpy effect, leading to the formation of a stable FCC structure. The grain size reduction and structural refinement of Al_2_O_3_ were studied using EBSD analysis.
❖ The hot-forging process helped to increase the relative density by approximately 21% compared to the hot-compacted samples owing to grain growth and decrease in yield strength at elevated temperatures.
❖ With the increase in the Al_2_O_3_ content, the hardness and mechanical strength gradually increased and the VHS and UCS of the CrFeCuMnNi-3 vol.% Al_2_O_3_ HEAMC sample were around 1.21 times higher than those of the unreinforced CrFeCuMnNi HEA matrix.
❖ Moreover, the wear resistance of the CrFeCuMnNi-3 vol.% Al_2_O_3_ HEAMC sample steadily improved by 52.9% compared to the unreinforced one due to the lower domination of adhesive and abrasive mechanisms and increase in the oxide protective layer.

## Figures and Tables

**Table 1:** Chemical composition of CrFeCuMnNi-Al_2_O_3_ high-entropy alloy matrix composites (HEAMCs).

Sample (ID)	Cr		Fe		Cu		Mn		Ni		Al_2_O_3_	
	at.%	wt.%	at.%	wt.%	at.%	wt.%	at.%	wt.%	at.%	wt.%	at.%	wt.%
CrFeCuMnNi-0 A_2_O_3_ (HEAMC-0A)	0.200	0.196	0.200	0.182	0.200	0.223	0.200	0.193	0.200	0.206	0.000	0.000
CrFeCuMnNi-1A_2_O_3_ (HEAMC-1A)	0.198	0.193	0.198	0.179	0.198	0.219	0.198	0.189	0.198	0.202	0.010	0.018
CrFeCuMnNi-2A_2_O_3_ (HEAMC-2A)	0.196	0.189	0.196	0.176	0.196	0.215	0.196	0.186	0.196	0.199	0.020	0.035
CrFeCuMnNi-3A_2_O_3_ (HEAMC-3A)	0.194	0.186	0.194	0.173	0.194	0.211	0.194	0.186	0.194	0.195	0.030	0.052

**Table 2:** Crystal structure, atomic radius, and physical properties of the developed HEAMCs.

Name of Element	Atomic Radius, pm	Crystal Structure	Melting Point (K)	Theoretical Density, kg/m^3^
Cr	140	BCC	1811	7140
Fe	140	BCC	2180	7874
Cu	135	FCC	1357	8920
Mn	140	FCC	1519	7260
Ni	135	FCC	1728	8908
Al_2_O_3_	-	Rhombohedral	2345	3987

**Table 3:** Structural parameters (peak position, FWHM, peak area, d-spacing, and peak height) of the hot-forged HEAMCs obtained from the XRD results.

Sample	Peak Position, 2θ (deg)	FWHM, deg	Peak Area	d-Spacing [Å]	Maximum Peak Height [cts]
CrFeCuMnNi	44.973	0.34158	379.325	2.01570	965
CrFeCuMnNi-1 vol.% Al_2_O_3_	44.1652	0.3657	246.28	2.05068	788.36
CrFeCuMnNi-2 vol.% Al_2_O_3_	43.5311	0.4093	307.34	2.07907	868.3256
CrFeCuMnNi-3 vol.% Al_2_O_3_	43.4662	0.4196	248.36	2.08202	1014

**Table 4:** Magnitude of the enthalpy mix ($H_{mix}^{AB}$, kJ/mol) calculated using Miedema’s model for the incorporated metallic elements.

	Fe	Cr	Cu	Mn	Ni
Fe	Fe	−1	13	0	−2
Cr	−1	Cr	12	2	−7
Cu	13	12	Cu	4	4
Mn	0	2	4	Mn	−8
Ni	−2	−7	4	−8	Ni

**Table 5:** Calculated thermodynamic parameters values (*δ*H_mix_, *δ*S_mix_, *δ*G_mix_, Ω, r, VEC, and *δ*X) for the developed ODS-HECs.

	δH_mix,_ kJ/mol	δS_mix_ J/mol.k	δG_mix_ J/mol	Ω	r, nm	δ, %	VEC	δX	Expected Phases
CrFeCuMnNi	2.72	13.38	−1267.5	8.4565	0.138	1.77	8.400	0.1418	FCC
CrFeCuMnNi−1 vol.%Al_2_O_3_	2.67	13.71	−1420.5	8.8743	0.136	2.05	8.316	0.1422	FCC
CrFeCuMnNi−2 vol.%Al_2_O_3_	2.61	13.93	−1538.36	9.2322	0.135	2.70	8.232	0.1430	FCC
CrFeCuMnNi−3 vol.%Al_2_O_3_	2.56	14.10	−1642.46	9.5739	0.133	3.54	8.148	0.15	FCC

**Table 6:** Mechanical properties in terms of relative density, ultimate compressive strength, strain at ultimate point, and the Vickers hardness strength of the developed HEAMCs.

Name of Sample	Relative Density at 550 Mpa Compaction Pressure	Relative Density at 1100 Followd Forging	Ultimate Compressive Stress, Mpa	Strain at Ultimate Point	Vickers Hardness Strength, Mpa
CrFeCuMnNi	0.6902 ± 0.0057	0.8295 ± 0.0124	872.3256 ± 5.3689	0.1388 ± 0.009	2648.3650 ± 7.3659
CrFeCuMnNi-1 vol.% Al2O3	0.6786 ± 0.0024	0.8137 ± 0.0066	954.0280 ± 3.5698	0.1072 ± 0.0057	2895.4560 ± 5.369
CrFeCuMnNi-2 vol.% Al2O3	0.6643 ± 0.0036	0.8027 ± 0.0014	1025.5140 ± 4.897	0.0857 ± 0.0065	3108.2350 ± 7.236
CrFeCuMnNi-3 vol.% Al2O3	0.6525 ± 0.0019	0.7957 ± 0.0115	1058.0670 ± 3.4780	0.0843 ± 0.0037	3203.5600 ± 5.698

## Data Availability

The experimental datasets obtained from this research work and the analysed results during the current study are available from the corresponding author upon reasonable request.
